# Lower diet quality accelerates DNA methylation-based age

**DOI:** 10.1007/s11357-025-01835-y

**Published:** 2025-08-15

**Authors:** Botong Shen, Nicole Noren Hooten, Nicolle A. Mode, Marie Fanelli Kuczmarski, Alan B. Zonderman, Michele K. Evans

**Affiliations:** Laboratory of Epidemiology and Population Sciences, National Institute On Aging, National Institutes of Health, 251 Bayview Boulevard Suite 100 Room 4C-222, Baltimore, MD 21224 USA

**Keywords:** DNA methylation, Race, Health disparities, Minority health, Inflammation, Diet quality, Diet index, Dietary score

## Abstract

A new DNA methylation biomarker, Dunedin Pace of Aging Calculated from the Epigenome (DunedinPACE), is associated with healthy lifespan in several European ancestry cohorts. Few studies have examined the relation between dietary quality and DunedinPACE in African American and White adults with longitudinal assessments. To assess the relationship between diet quality and DunedinPACE, we used longitudinal data from African American and White 30–64 year old adults living above and below poverty. Participants’ DunedinPACE scores and dietary assessments were calculated at two time points, approximately 5 years apart. Numbers of participants (*n* = 421; mean age 49 years) were balanced by race, sex, and poverty status. Diet quality was assessed using two different dietary indexes: Dietary Inflammatory Index (DII) and Healthy Eating Index-2010 (HEI). Linear mixed model regression examined the longitudinal association of DunedinPACE with DII and HEI adjusted by age, race, poverty status, BMI, and smoking status. Initial mean values of DII were 3.34 (SD = 2.16) and HEI was 40.67 (SD = 11.69), indicating a pro-inflammatory dietary pattern and low diet quality in this cohort. The initial mean DunedinPACE score was 1.07. We found that a higher DII score was associated with higher DunedinPACE score (β = 0.009; *p* < 0.001), higher HEI score was associated with lower DunedinPACE score (β =  − 0.001; *p* = 0.032), and that these relationships were consistent over time. Overall, lower dietary quality was associated with a faster pace of aging captured by DunedinPACE score. Our findings demonstrate the independent contribution of diet quality to healthy aging-related epigenetic mechanisms.

**Graphical Abstract:**

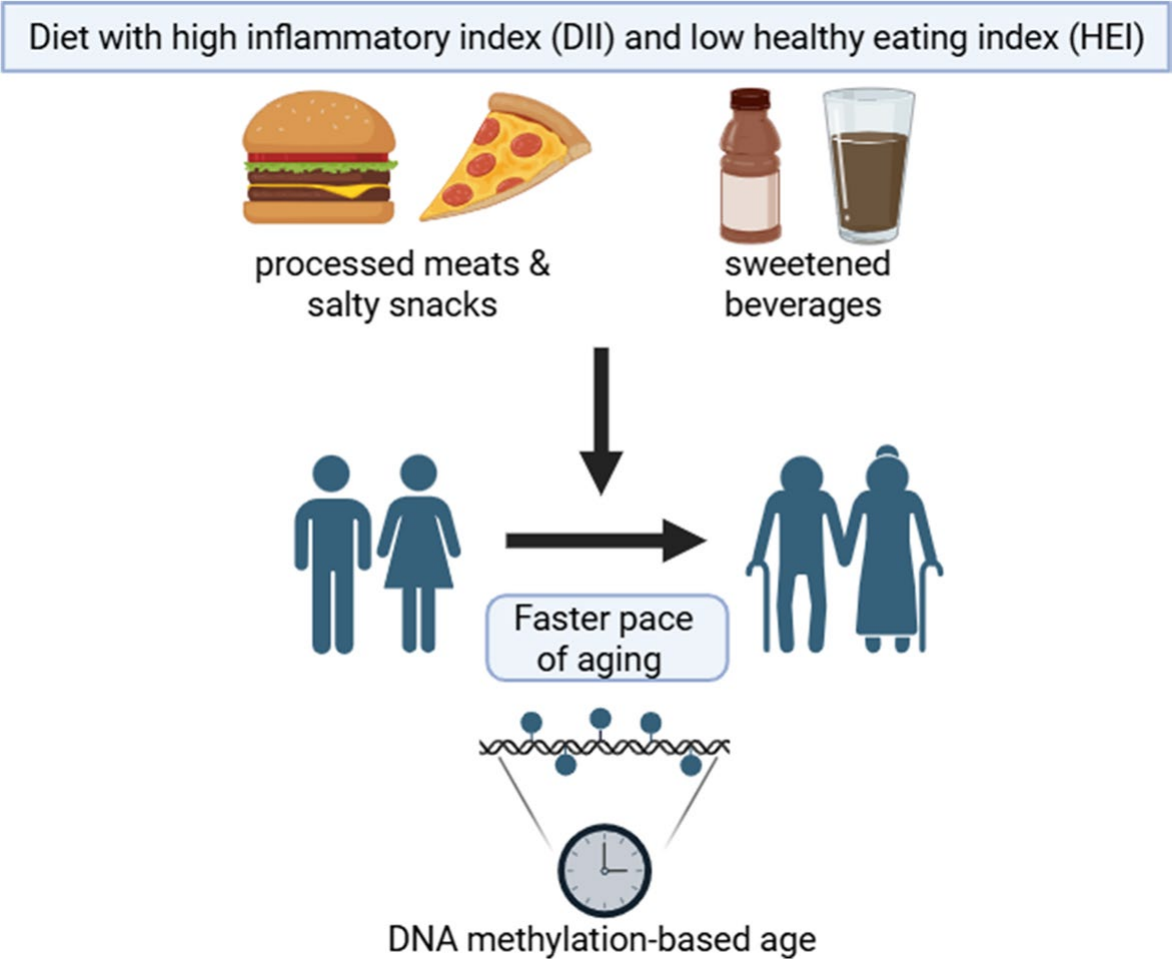

## Introduction

Diet quality is associated with age-associated disease and organismal lifespan [1, 2]. Dietary patterns at midlife can have consequences later in life including reducing risk for age-associated disease and conditions such as Alzheimer’s disease, frailty, and cardiovascular disease (reviewed in [2]). Understanding how diet and nutrition affect the aging process is key to improving health and longevity.

A high-quality diet reflects a healthy pattern of consumption providing optimal intakes of nutrients and dietary constituents, like flavonoids, that are associated with lower disease risk. There are many ways to measure diet quality*.* A priori analytical approaches to measure diet quality typically score food intake patterns in terms of how closely they align with national dietary guidelines, such as the Healthy Eating Index (HEI), or in terms of their adherence to a particular cuisine such as the Mediterranean Dietary Score. The HEI-2010 (hereafter referred to as HEI) assesses diet quality from two perspectives—adequacy (nine dietary components to increase) and moderation (three dietary components to decrease) and uses scoring standards that are density-based [3]. The HEI measures diet quality conformance to the 2010 Dietary Guidelines for Americans (DGA [4]) with higher scores indicating closer compliance with US dietary guidance. In contrast, the Dietary Inflammatory Index (DII) was created to estimate the inflammatory potential of the diet in any human population [5]. The DII is based on literature from a variety of different study designs ranging from cell cultures to observational and experimental studies with humans from around the world, as well as evidence from qualifying laboratory animal experiments. Low scores indicate anti-inflammatory potential of the diet while high scores reflect pro-inflammatory potential. In young adults, DII was negatively correlated with the HEI-2010 (*r* =  − 0.65, *p* < 0.01) and other diet quality indices, namely the Dietary Approaches to Stop Hypertension (*r* =  − 0.52, *p* < 0.01) and the Alternative HEI (*r* =  − 0.55, *p* < 0.01) [6].

Recent data indicate that diet may influence aging through regulating epigenetic mechanisms, including alterations in DNA methylation. DNA methylation (DNAm) at CpG sites are modified with age and are often used as biological indicators of aging, called epigenetic age [7–9]. Several different epigenetic age estimators have been utilized including the first-generation clocks by Horvath [10] and Hannum [11] and second-generation clocks DNAm PhenoAge [12] and the DNAm GrimAge [13]. Third-generation clocks were developed including the initial Dunedin Pace of Aging Methylation (DunedinPoAm) [14] and the most recent Dunedin Pace of Aging Calculated From the Epigenome (DunedinPACE) measure [15]. The DunedinPACE measure was designed to assess the pace of aging by using 20 years of longitudinal measurements of 19 different biomarkers of system-integrity in the Dunedin Birth Cohort Study [15].

The DunedinPACE epigenetic age estimator appears to be able to capture influences of lifestyle and diet on the pace of aging. For example, data from the Comprehensive Assessment of Long-term Effects of Reducing Intake of Energy (CALERIE) trial found that calorie restriction slowed the pace of aging as measured by DunedinPACE but not with the second-generation clocks [16]. However, we are only beginning to understand how diet interventions may affect epigenetic age. Data from the DIRECT-PLUS (dietary intervention randomized controlled trial polypenols-unprocessed) trial indicated that there were no differences in biological age, including the DunedinPACE measure and other measures, between different dietary interventions [17]. A cross-sectional study from the Framingham Offspring Cohort of older adults (mean age = 67 years) found that a higher Dietary Approaches to Stop Hypertension (DASH) score was associated with decreased biological age measures, DunedinPoAM, GrimAge, and PhenoAge [18]. Another cross-sectional study from postmenopausal women in the Women’s Health Initiative found that higher DASH, HEI-2015, and alternative Mediterranean diet (aMED) were negatively associated with DunedinPACE and other epigenetic measures [19]. Additionally, the Sister Study found that women consuming a healthy diet had lower epigenetic age as measured using first- and second-generation clocks [20]. In Black and White women (*n* = 342; mean age = 39.2 years) aMED, alternate HEI-2010, mean sugar intake and also a developed “Epigenetic Nutrient Index” based on nutrient-based approach, were associated with GrimAge2 [21]. Another recent study found a weak but significant negative relationship between HEI and DunedinPACE in healthy Hawaiian residents [22]. Therefore, there may be different relationships between diet and epigenetic age in different populations, indicating the importance of analyzing data from various populations and assessing multiple diet measures to further examine these relationships.

Analysis examining specific dietary components indicates that markers of fruit/vegetables are associated with lower DNAm PhenoAge [12] and DNAm GrimAge [13] and higher fat consumption was associated with greater DNAm GrimAge [13]. Using first-generation clocks, Quach et al. reported that fish, poultry, and fruits/vegetables consumption were associated with epigenetic age [23]. Focusing on specific dietary components limits how overall diets may affect epigenetic age. In addition, as most existing diet and epigenetic studies are cross-sectional, there is a need to incorporate longitudinal analyses.

Here we analyzed the association of the pace of aging using the DunedinPACE measure with diet quality using two different dietary indexes in a middle-aged cohort of African American and White adults living above and below poverty. Few studies incorporate multiple population groups in nutritional as well as epigenetic aging studies. We utilized longitudinal measures of DNAm and diet to assess how diet quality affects the pace of aging using data from the HANDLS study.

## Methods

### Study population

**Fig. 1:**
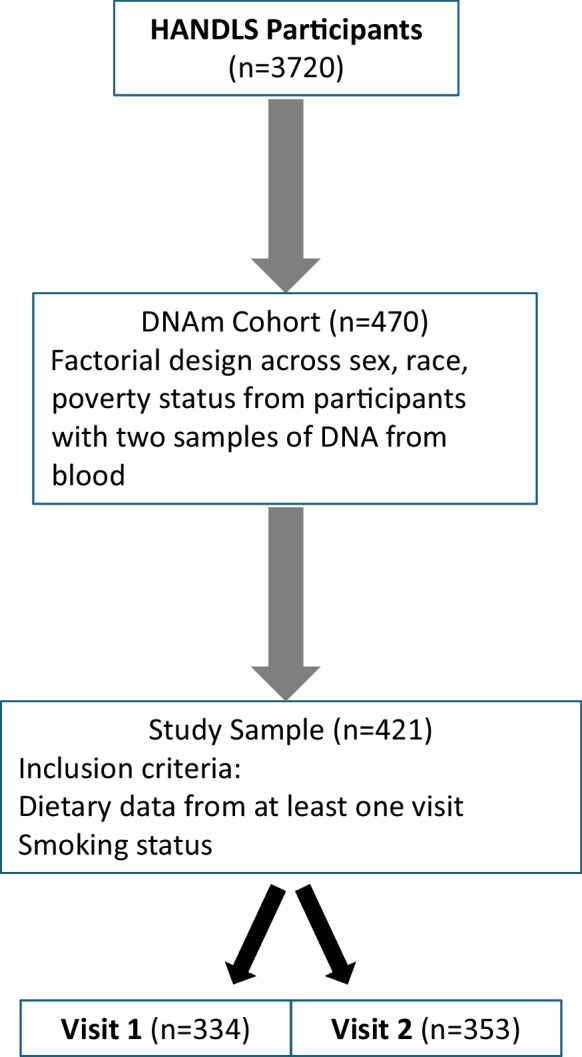
Flow chart for sample selection

Participants were selected from HANDLS, a prospective population-based longitudinal study with a fixed cohort of community-dwelling African American and White participants [24] in Baltimore, Maryland. HANDLS was initiated in 2004 with men and women aged initially between 30 and 64 years. At enrollment, participants self-identified as either African American or White. Poverty status was defined as above or below 125% of the 2004 US federal poverty guidelines for household income [25]. Sex was sex assigned at birth. Time 1 examination data were collected through a home visit and examination on medical research vehicles between August 14, 2004, and June 22, 2009; participants were administered a physical health examination, medical history inquiries, two repeated 24-h dietary recalls, and other assessments. Time 2 data were collected between June 23, 2009, and September 12, 2017; follow-up in-person visits were conducted for participants using protocols similar to time 1. The HANDLS study protocol was approved by the Institutional Review Board of the National Institutes of Health. All participants provided written informed consent. Participants for the current study (Fig. 1) came from a DNAm cohort randomly selected using a factorial design of sex, race, and poverty status from HANDLS participants with blood samples at time 1 and time 2 [26, 27]. The sample in this study further required dietary data, body mass index (BMI; kg/m^2^) from measured height and weight, and smoking status from the medical history (ever/never), resulting in 421 participants with at least one visit across time 1 (*n* = 334) and time 2 (*n* = 353). The average follow-up time was 5.1 years between time 1 and time 2.

### DNA methylation measures

DNA methylation in peripheral white blood cell samples was profiled using the Illumina Human MethylationEPIC BeadChip as described previously [27]. The β-value for each CpG site was used for DNAm level measures, adjusted for batch effects, and white blood cell proportions. The white blood cell proportions of each cell type were estimated using Houseman’s method [28]. Low-quality samples and β-value outliers were excluded. For each participant, DunedinPACE scores were calculated at two time points based on the β-values using R package DunedinPACE developed by Belsky et al. (4).

### Dietary inflammatory index

For HANDLS participants, two nonconsecutive 24-h dietary recalls were administered to collect food and beverage intake information. Data were collected by trained interviewers using the Automated Multiple-Pass Method (AMPM) established by the United States Department of Agriculture [29]. The DII was calculated for each recall day using parameters defined by Shivappa et al. [30]. The parameters included energy, alcohol, protein, carbohydrate, dietary fiber, total fat, saturated fat, monounsaturated fat, polyunsaturated fat, omega 3 fatty acids, omega 6 fatty acids, cholesterol, 11 vitamins, 4 minerals, 6 flavonoid classes, caffeine, and tea. The DII scores were standardized to global referent values [30]. Final DII values are the mean across the 2 recall days. The possible maximal pro-inflammatory DII score for the HANDLS study sample was + 10.44 and the maximal anti-inflammatory DII score was − 10.44.

### Healthy eating index

The score for each of the 12 components of the HEI was calculated using the procedure provided on The National Cancer Institute’s Applied Research website [31]. The nine adequacy components included total fruits, whole fruits, total vegetables, greens and beans, whole grains, dairy, total protein foods, seafood and plant proteins, and fatty acids. The three moderation components included refined grains, sodium, and empty calories (solid fats, added sugar, and alcohol). A detailed description for our calculation of HEI scores is available on the HANDLS website [24]. HEI scores were calculated for each recall day and averaged to obtain the mean. The maximum possible score was 100.

### Statistical analysis

Analyses at baseline consisted of tests between groups at time 1 using two-sided Student’s *t*-test for continuous variables and Pearson’s chi-square test for dichotomous variables. Correlation used Pearson’s correlation test. Longitudinal analyses were performed using linear mixed model regression to account for repeated measurements without excluding participants with only one time point of data. The model included a random intercept for each person. Chronological age (years) was included in the models as a fixed effect in decade units centered at age 50 ([age – 50]/10). Based on previous work [26], a fixed quadradic term for age was also included in the models. Each dietary measure (HEI, DII) was analyzed for its relationship with DunedinPACE in a separate regression model with the fixed factors of race, poverty status, age, quadradic age, BMI, and smoking status. Backward elimination was employed starting with three-way interactions which were examined and removed if not significant. Significance was defined as two-sided tests using *p* < 0.05. Analyses were performed using R version 4.4.1 [32]. Linear mixed model regression was done using the R package lmer, and beta coefficient *p*-values estimated using the R package lmerTest.

## Results

### Study sample characteristics at baseline

At time 1, the mean age for participants was 48.6 years (SD = 8.8) and ranged from 30.2 to 64.8 years (Table 1). Over half of the participants at time 1 reported having smoked in their lifetime (69.6%). There were no significant age differences at time 1 between participants with household incomes below or above poverty (*p* = 0.676), between African American and White participants (*p* = 0.702), or between men and women (*p* = 0.723). The mean DunedinPACE score at time 1 was 1.07 (SD = 0.14), indicating an aging rate 7% older than indicated by chronology. DunedinPACE scores and chronological age were positively correlated in the sample (Pearson correlation *r* = 0.16, *p* = 0.004). The study sample at time 1 had a mean DII of 3.39 (SD = 2.15) and mean HEI of 40.67 (SD = 11.69).

**Table 1:** Participant demographic characteristics and dietary indexes

Variable	*N* = 421	
Race, *n* (%)		
African American	210 (49.9)	
White	211 (50.1)	
Sex, *n* (%)		
Male	219 (52.0)	
Female	202 (48.0)	
Poverty Status, *n* (%)		
Above	212 (50.4)	
Below	209 (49.6)	
	Time 1 *N* = 334	Time 2 *N *= 353
Age, years mean (sd^1^)	48.6 (8.8)	53.8 (8.5)
DunedinPACE, mean (sd)	1.07 (0.1)	1.10 (0.1)
Dietary Inflammation Index, mean (sd)	3.4 (2.2)	3.4 (2.1)
Healthy Eating Index 2010, mean (sd)	40.7 (11.7)	44.9 (12.2)
BMI^2^, mean (sd)	29.8 (7.5)	30.7 (7.7)
Ever smoker, *n* (%)	229 (69.6)	255 (72.2)

^1^Standard deviation

^2^Body mass index, kg/m^2^

### Longitudinal association between DII and DunedinPACE

**Fig. 2:**
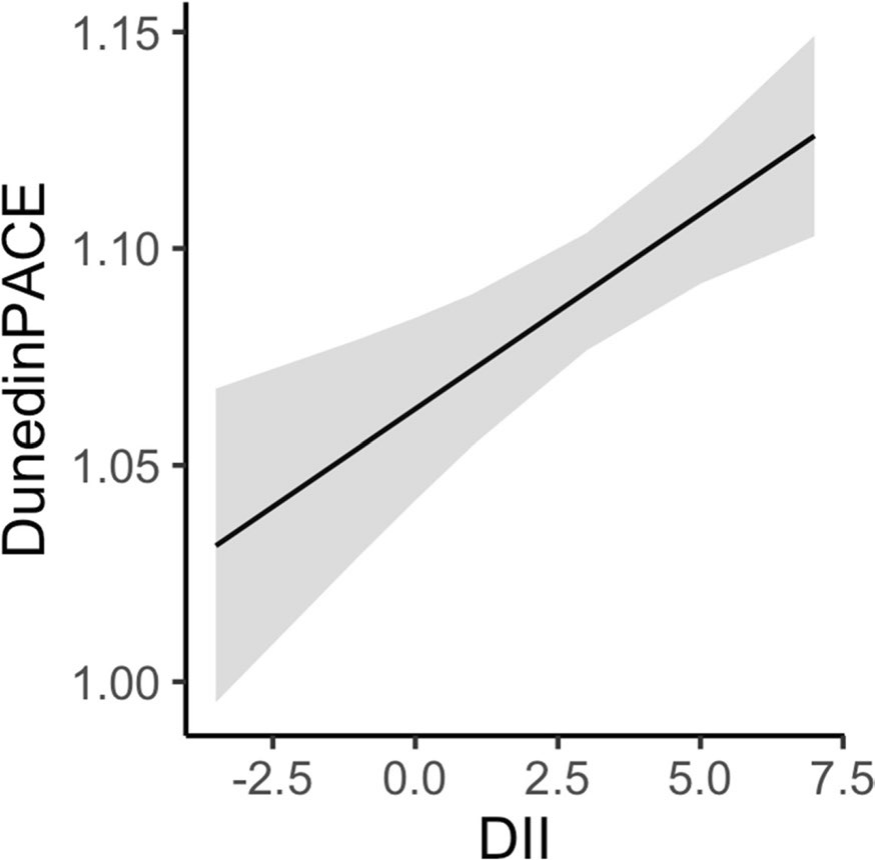
Relationship of DunedinPACE and Dietary Inflammation Index from linear mixed model regression at two time points. Linear mixed model regression was used to analyze the relationship of DunedinPACE and Dietary Inflammatory Index (DII) and included age, quadradic age, body mass index, ever smoked, and an interaction of race and poverty status as fixed effects and a random intercept for participant. Data included participants with DunedinPACE and dietary data at two time points (*n* = 421). Line from the regression model is shown for DII and DunedinPACE and gray indicates the 95% confidence interval. A higher DII indicates a pro-inflammatory diet

We examined the separate longitudinal relationships between DII and HEI with DunedinPACE. Linear mixed model regression revealed significant main effects for quadratic age (age^2^), BMI, smoking status, DII, and a significant two-way interaction between race and poverty status (Table 2). Participants with higher DII scores were associated with higher DunedinPACE score, indicating faster pace of aging (β = 0.009; *p* < 0.001; Fig. 2). A one standard deviation change in DII (2.2) corresponded to 2% faster pace of biological aging than chronological aging. Smoking and greater BMI were also associated with higher DunedinPACE score (Table 2). We confirmed our previous finding of the significant two-way interaction between race and poverty status that White adults living above poverty status had a significantly lower DunedinPACE score than White adults with household incomes below poverty, and African American adults either below or above poverty (*p* < 0.001) [26]. The other groups were not significantly different from one another. Sex was not significantly related to DunedinPACE scores in these analyses. There were no significant interactions with age indicating that the relationships between DunedinPACE and DII, as well as with race and poverty status, did not change over time within this study period.

**Table 2:** Linear mixed model regression analysis of association of DunedinPACE scores with dietary inflammatory index at two time points

Term	Beta coefficient	Standard error	*p*-value
Race (white)	− 0.040	0.015	0.008
Poverty Status (below)	− 0.011	0.015	0.448
Age^1^	0.010	0.006	0.080
Age (quadradic)	− 0.011	0.006	0.046
BMI^2^	0.001	0.001	0.032
Ever smoker	0.042	0.012	< 0.001
DII^3^	0.009	0.003	< 0.001
Race × poverty status	0.051	0.021	0.015

^1^Age is in decade units, centered at 50 ((age – 50)/10)

^2^Body mass index, kg/m^2^

^3^Dietary Inflammatory Index: lower values indicate better diet quality

### Longitudinal association between HEI and DunedinPACE

**Fig. 3:**
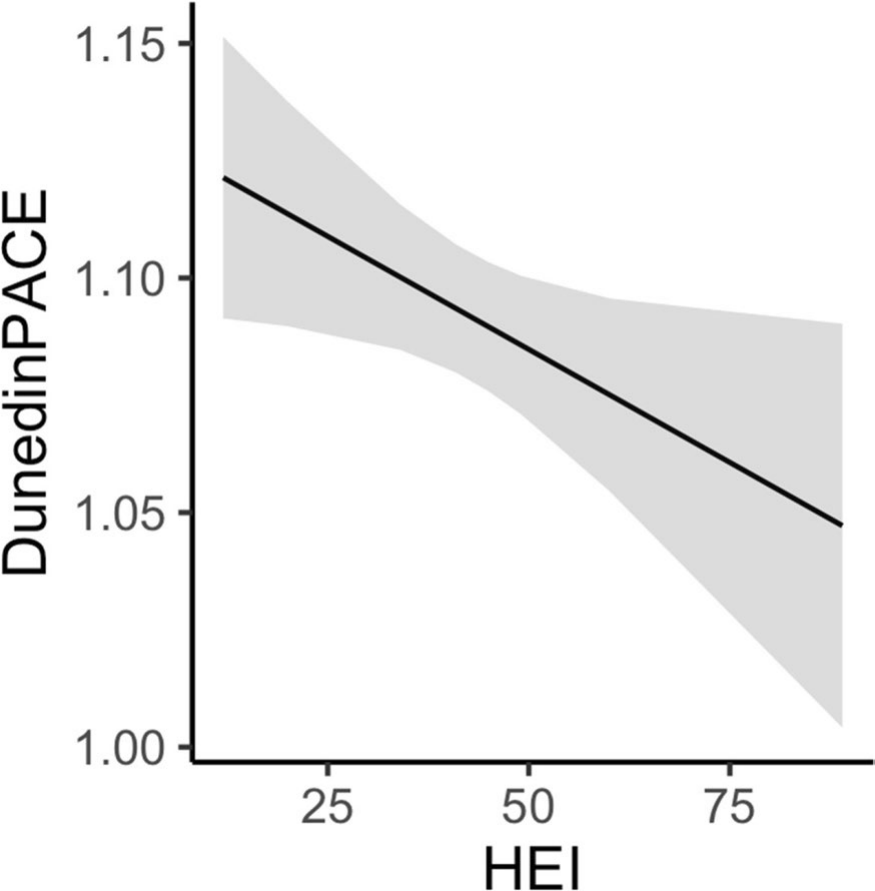
Relationship of DunedinPACE and Healthy Eating Index from linear mixed model regression at two time points Linear mixed model regression was used to analyze the relationship of DunedinPACE and Healthy Eating Index (HEI) and included age, quadradic age, body mass index, ever smoked, and an interaction of race and poverty status as fixed effects and a random intercept for participant. Data included participants with DunedinPACE and dietary data at two time points (*n* = 421). Line from the regression model is shown for HEI and DunedinPACE and gray indicates the 95% confidence interval. A higher HEI score indicates a healthier diet

Linear mixed model regression revealed significant main effects for quadratic age (age^2^), BMI, smoking status, HEI, and a significant two-way interaction between race and poverty status (Table 3). Participants with higher HEI scores, indicating diets of better quality and greater compliance to the Dietary Guidelines for Americans, were associated with lower DunedinPACE scores, indicating slower pace of aging (β =  − 0.001; *p* = 0.032; Fig. 3). A one standard deviation change in HEI (11.7) corresponded to a 1% slower pace of biological aging than chronological aging. We observed similar associations for BMI, smoking, and the two-way interaction between race and poverty status as in the DII analysis. Sex was not significantly related to DunedinPACE scores in these analyses. There were no significant interactions with age indicating that the relationships between DunedinPACE and HEI did not change over time within this study period.

**Table 3:** Linear mixed model regression analysis of association of DunedinPACE scores with healthy eating index at two time points

Term	Beta coefficient	Standard error	*p*-value
Race (White)	− 0.049	0.015	0.001
Poverty Status (below)	− 0.012	0.015	0.407
Age^1^	0.012	0.006	0.051
Age (quadradic)	− 0.011	0.006	0.045
BMI^2^	0.002	0.001	0.018
Ever smoker	0.038	0.012	0.002
HEI^3^	− 0.001	0.0004	0.032
Race × poverty status	0.058	0.021	0.006

^1^Age is in decade units, centered at 50 ((age – 50)/10)

^2^Body mass index, kg/m^2^

^3^Healthy Eating Index 2010: higher values indicate better diet quality

## Discussion

In a cohort of African American and White adults living above or below poverty, we found that lower diet quality was associated with a faster pace of aging. We also utilized longitudinal nutritional and epigenetic data. These data build upon earlier studies that have focused on specific dietary components and first- or second-generation epigenetic measures [12, 13, 23, 33]. These studies found that specific dietary components, those considered healthy food options including fruits/vegetables and fish intake, are associated with lower epigenetic age whereas red meat intake accelerates epigenetic age. Our data support these findings.

In this study, we utilized the DII and HEI, two measures of diet quality which are indicators of different aspects of dietary consumption. Therefore, we not only acquire data on conformance to the DGA but also on the inflammatory potential of the diet. Other studies have examined the DASH diet [18] and other nutritional indexes (DASH, aMED, Alternative HEI-2010 or HEI-2015) [19–21] and epigenetic age estimators. Our data are consistent with the findings in these reports that healthy diets are associated with lower epigenetic age. However, here we have used longitudinal data to analyze the relationship between two different dietary indexes and the pace of aging, using the newest epigenetic measure DunedinPACE. Interestingly in the Framingham Heart Study Offspring cohort, a mediation analysis found that DNAm may partially explain the relationship between diet (as measured using DASH) and mortality [18]. Consistent with this finding, hypermethylation of cg18181703 (*SOCS3*) was found to be associated with higher diet quality (Mediterranean-style diet score and Alternative HEI) and a reduced risk for all-cause mortality in a European ancestry cohort [34]. These data point to DNA methylation changes as a potential mediator between diet and mortality.

Here we found evidence that a higher inflammatory potential of the diet may contribute to a faster pace of aging. A pro-inflammatory diet may lead to inflammation, which is a well-known driver of aging and age-related diseases [35, 36]. Specific circulating inflammatory biomarkers have previously been associated with epigenetic age acceleration [13, 37]. Taken together, these data indicate that eating a diet with pro-inflammatory potential may lead to DNAm changes that promote chronic inflammation and an accelerated aging phenotype.

Both a strength and a limitation to our study is the fact that our cohort overall has a low diet quality and pro-inflammatory dietary pattern. The HEI-2010 scores were approximately 12–17 points lower than those of a representative US sample (HEI Scores for Americans. https://www.fns.usda.gov/cnpp/hei-scores-americans, Updated 4/28/2022). One strength is that we utilized longitudinal data of both African American and White adults living above and below poverty that are often not included in diet and epigenetic studies. At the two different visits, data were collected on separate days using 24-h recalls, ensuring data from both weekday and weekend days. We used the AMPM, an accurate validated method, to collect dietary data [29]. However, it should be noted that self-reporting of dietary information is subject to bias based on social desirability [38]. Another strength is that we assessed multiple aspects of diet quality through the DII and HEI. One limitation is that our data were collected over two visits approximately 5 years apart, which may be too short of a time period in a middle-aged cohort. Collecting both DNAm and dietary information at additional time points will be important to further decipher the role of diet on epigenetics over the lifespan. The effect sizes that we reported are significant but fairly small. Nonetheless, it has been reported that lifestyle factors at midlife are known to contribute to health outcomes later in life [39, 40]. Therefore, modifying factors, e.g., eating a healthy diet, at midlife may have long-term consequences for healthy aging.

In this longitudinal cohort study, our findings revealed lower dietary quality was associated with a higher DundinPACE score. These results support that eating a low quality diet may accelerate the biologic pace of aging as measured using the DunedinPACE DNAm biomarker. Consequently, these data indicate that eating a healthy, anti-inflammatory diet can promote healthy aging.

## Data Availability

Data described in the manuscript, code book, and analytic code will be made available pending reasonable request through the HANDLS website https://handls.nih.gov/.

## Abbreviations

DASH: Dietary Approaches to Stop Hypertension
DNAm: DNA methylation
DGA: Dietary Guidelines for Americans
DII: Dietary inflammatory index
DunedinPACE: Pace of Aging Calculated from the Epigenome
HANDLS: Healthy Aging in Neighborhoods of Diversity across the Life Span
HEI: Healthy eating index

## Declarations

### Data sharing

Data described in the manuscript, code book, and analytic code will be made available pending reasonable request through the HANDLS website https://handls.nih.gov/.
